# Hexaaqua­magnesium(II) bis­{[*N*-(4-meth­oxy-2-oxidobenzyl­idene)glycyl­glycinato(3−)]cuprate(II)} hexa­hydrate

**DOI:** 10.1107/S1600536809040872

**Published:** 2009-10-13

**Authors:** Jiaxun Jiang, Yao Lu, Limin Yuan, Wenlong Liu

**Affiliations:** a College of Chemistry and Chemical Engineering, Yangzhou Universitry, Yangzhou 225002, People’s Republic of China

## Abstract

In the title complex, [Mg(H_2_O)_6_][Cu(C_12_H_11_N_2_O_5_)]_2_·6H_2_O, the Cu^II^ atoms lie at the center of the square plane of triple negatively charged *O*,*N*,*N*′,*O*′-tetra­dentate Schiff base ligands, which are coordinated by one phenolate O atom, one imine N atom, one deprotonated amide N atom and one carboxyl­ate O atom. The Mg^II^ center, which sits on an inversion center, is coordinated by six aqua ligands and exhibits a slightly distorted octa­hedral conformation. The asymmetric unit consists of an [*N*-(4-meth­oxy-2-oxidobenzyl­idene)glycyl­glycinato]cuprate(II) anion, one half of an [Mg(H_2_O)_6_]^2+^ cation and three free water mol­ecules. The cations and anions form columns by O—H⋯O hydrogen bonds.

## Related literature

For structures of Schiff base analogues, see: Gupta *et al.* (2009 ▶); Vigato *et al.* (2007 ▶). For structures of Schiff base heteronuclear complexes, see: Jiang *et al.* (2009 ▶); Sakamoto *et al.* (2001 ▶); Vigato & Tamburini (2008 ▶); Zhang *et al.* (2008 ▶).
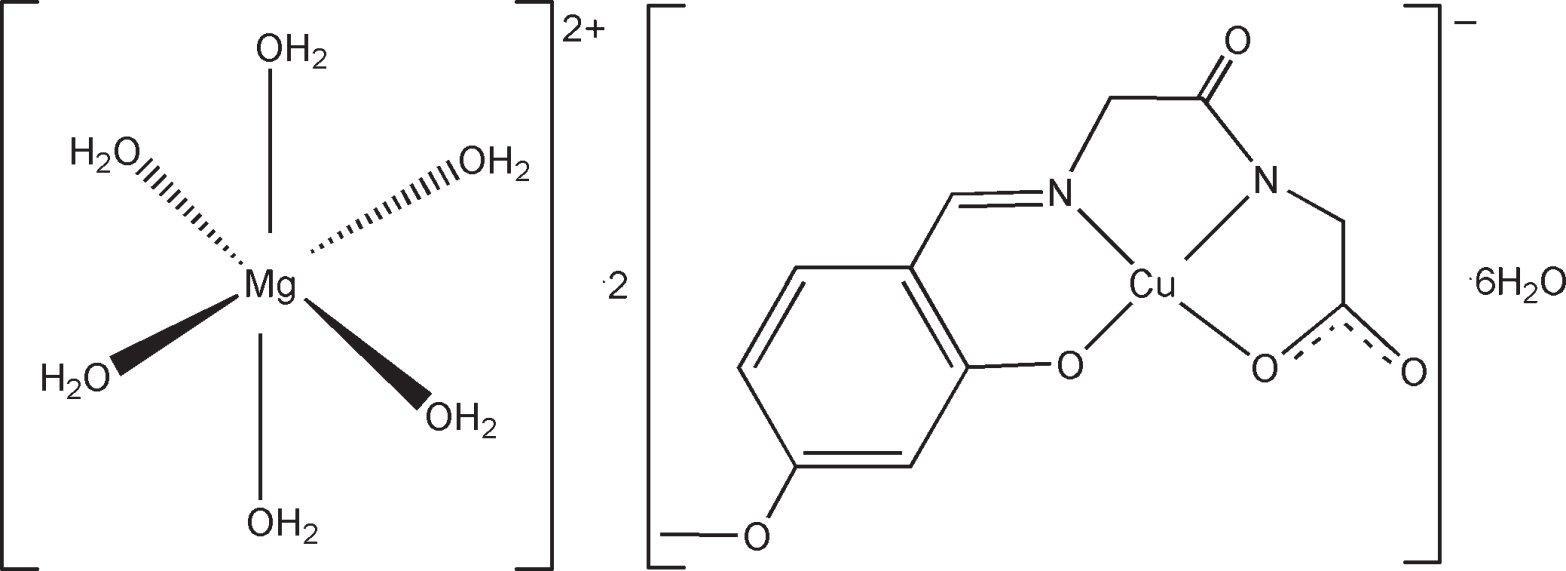


## Experimental

### 

#### Crystal data

[Mg(H_2_O)_6_][Cu(C_12_H_11_N_2_O_5_)]_2_·6H_2_O
*M* *_r_* = 894.04
Triclinic, 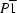

*a* = 7.8606 (14) Å
*b* = 10.933 (2) Å
*c* = 11.539 (2) Å
α = 76.650 (2)°
β = 76.685 (2)°
γ = 80.737 (2)°
*V* = 932.8 (3) Å^3^
*Z* = 1
Mo *K*α radiation
μ = 1.25 mm^−1^
*T* = 296 K
0.30 × 0.28 × 0.25 mm

#### Data collection

Bruker SMART APEX CCD diffractometer
Absorption correction: multi-scan (*SADABS*; Sheldrick, 2004 ▶) *T*_min_ = 0.696, *T*_max_ = 0.736
6808 measured reflections
3262 independent reflections
2836 reflections with *I* > 2σ(*I*)
*R*_int_ = 0.084

#### Refinement

*R*[*F* ^2^ > 2σ(*F* ^2^)] = 0.036
*wR*(*F* ^2^) = 0.097
*S* = 1.04
3262 reflections
278 parameters
18 restraints
H atoms treated by a mixture of independent and constrained refinement
Δρ_max_ = 0.76 e Å^−3^
Δρ_min_ = −0.57 e Å^−3^

### 

Data collection: *SMART* (Bruker, 2002 ▶); cell refinement: *SAINT-Plus* (Bruker, 2003 ▶); data reduction: *SAINT-Plus*; program(s) used to solve structure: *SHELXTL* (Sheldrick, 2008 ▶); program(s) used to refine structure: *SHELXTL*; molecular graphics: *SHELXTL* and *DIAMOND* (Brandenburg, 2006 ▶); software used to prepare material for publication: *SHELXTL*.

## Supplementary Material

Crystal structure: contains datablocks I, global. DOI: 10.1107/S1600536809040872/zq2010sup1.cif

Structure factors: contains datablocks I. DOI: 10.1107/S1600536809040872/zq2010Isup2.hkl

Additional supplementary materials:  crystallographic information; 3D view; checkCIF report

## Figures and Tables

**Table 1:** Hydrogen-bond geometry (Å, °)

*D*—H⋯*A*	*D*—H	H⋯*A*	*D*⋯*A*	*D*—H⋯*A*
O6—H6*B*⋯O4	0.851 (17)	1.907 (18)	2.755 (3)	175 (3)
O6—H6*A*⋯O2^i^	0.840 (17)	2.016 (18)	2.836 (2)	165 (3)
O7—H7*A*⋯O10^ii^	0.840 (17)	1.91 (2)	2.734 (3)	165 (3)
O7—H7*B*⋯O9^ii^	0.833 (17)	1.959 (18)	2.776 (3)	166 (3)
O8—H8*C*⋯O11	0.831 (17)	1.978 (18)	2.797 (3)	169 (3)
O8—H8*D*⋯O3	0.822 (17)	1.957 (17)	2.775 (2)	173 (3)
O9—H9*A*⋯O10^iii^	0.848 (18)	1.985 (19)	2.787 (3)	157 (3)
O9—H9*B*⋯O1	0.810 (18)	2.009 (19)	2.816 (3)	174 (4)
O10—H10*C*⋯O2^iv^	0.826 (18)	1.986 (19)	2.805 (3)	171 (4)
O10—H10*D*⋯O4^ii^	0.810 (17)	2.049 (18)	2.857 (3)	176 (4)
O11—H11*A*⋯O9^v^	0.813 (18)	2.050 (19)	2.857 (3)	172 (4)
O11—H11*B*⋯O2^vi^	0.835 (18)	2.101 (19)	2.927 (3)	170 (4)

Symmetry codes: (i) 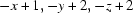; (ii) 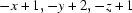; (iii) 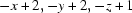; (iv) 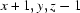; (v) 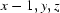; (vi) 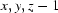.

**Fig. 1.**
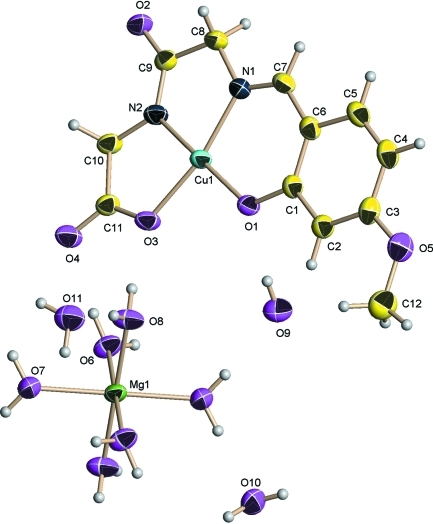
The molecular structure of (I), with atom labels and 50% probability displacement ellipsoids. Unlabeled atoms are related to labeled atoms by the symmetry code (-x + 1, -y + 2, -z + 1).

**Fig. 2.**
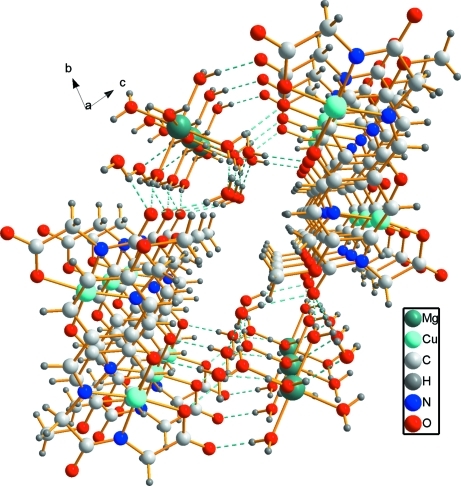
A packing diagram of (I), viewed down the a-axis, showing a separated column stacking structure connected by O—H···O hydrogen bonds (dashed lines).

## supplementary crystallographic information

### Comment

The Schiff bases are widely employed as ligands in coordination chemistry. These ligands are readily available, versatile, they exhibit various denticities and functionalities (Vigato *et al.*, 2007; Gupta *et al.*, 2009). Moreover, the number, the nature, and the relative position of the donor atoms of a Schiff base ligand allow a good control over the stereochemistry of the metallic centers, as well as over the number of the metal ions within homo- and heteronuclear complexes (Vigato *et al.*, 2008; Sakamoto *et al.*, 2001). Now we report the synthesis and structure of Cu^II^—Mg^II^ Schiff base complex derived from glycylglycine and 4-methoxy-salicylaldehyde.

The heteronuclear complex (I) crystallizes in the triclinic space group *P*1. The asymmetric unit consists of one [CuL]^-^ anion (*L* is a Schiff base derived from glycylglycine and 4-methoxy-salicylaldehyde), one half of the Mg(H_2_O)_6_^2+^ cation [Mg1, O6, O7, O8] and three uncoordinated water molecules [O9, O10, O11] in the complex (I) (Fig. 1). The deprotonated Schiff base is a triple negatively charged tetradentate ONNO ligand, coordinating to the Cu^II^ atom by one phenolate O atom [O1] (Cu1—O1 = 1.880 (2) Å), one imine N atom [N1] (Cu1—N1 = 1.920 (2) Å), one deprotonated amide N atom [N2] (Cu1—N2 = 1.892 (2) Å) and one carboxylato O atom [O3] (Cu1—O3 = 1.980 (2) Å). [CuL]^-^ exhibits approximately a square-planar structure. The Cu^II^ atom is in a slightly distorted square-planar environment with four donor atoms deviating from their mean plane by -0.0506 (9) Å (N1), +0.0626 (9) Å (N2), +0.0513 (8) Å (O1) and -0.0496 (9) Å (O3) (observed bond angles vary from 83.5 (1)° to 96.9 (1)°). The benzene ring [C1–C6] and the chelate ring [O1, C1, C6, C7, N1, Cu1] are almost coplanar with a dihedral angle of 0.11 (9)°, suggesting a large π-electron delocalization. The Mg^II^ atom lies on an inversion center and the coordination by six aqua ligands exhibits a slightly distorted octahedral environment. The six Mg—O bonds in the structure are in the range of 2.059 (2) - 2.063 (2) Å. In the crystal structure, the [CuL]^-^ anions and [Mg(H_2_O)_6_]^2+^ cations each form columns by hydrogen bonds along the *a*-axis (Fig. 2, Table 1).

### Experimental

Glycylglycine (5 mmol), 4-methoxy-salicylaldehyde (5 mmol) and NaOH (10 mmol) were dissolved in MeOH/H_2_O (30 ml, v: v = 1: 1) and refluxed for 30 min. Then Cu(ClO_4_)_2_.6H_2_O (5 mmol) was added to the solution and the resulting solution was adjusted to 9–11 by 5 mol/*L* NaOH solution. After stirring at room temperature for another 1 hr, MgCl_2_.6H_2_O (2.5 mmol) was added. A violet precipitate was obtained immediately. After stirring for 30 min and then filtered, the precipitate was recrystallized in water. The violet crystals of complex (I) suitable for an X-ray diffraction analyses were obtained after 1 week.

### Refinement

The water H atoms were located in a difference Fourier map and refined with restraints: O—H = 0.85 Å and *U*_iso_(H) =1.5*U*_eq_(O). All other H atoms were positioned geometrically and constrained as riding atoms, with C—H distances of 0.93–0.97 Å and *U*_iso_(H) set to 1.2 or 1.5*U*_eq_(C) of the parent atom.

### Crystal data

**Table:**

[Mg(H_2_O)_6_][Cu(C_12_H_11_N_2_O_5_)]_2_·6H_2_O	*Z* = 1
*M**_r_* = 894.04	*F*(000) = 464
Triclinic, *P*1	*D*_x_ = 1.592 Mg m^−^^3^
Hall symbol: -P 1	Mo *K*α radiation, λ = 0.71073 Å
*a* = 7.8606 (14) Å	Cell parameters from 7186 reflections
*b* = 10.933 (2) Å	θ = 1.0–28.3°
*c* = 11.539 (2) Å	µ = 1.25 mm^−^^1^
α = 76.650 (2)°	*T* = 296 K
β = 76.685 (2)°	Block, violet
γ = 80.737 (2)°	0.30 × 0.28 × 0.25 mm
*V* = 932.8 (3) Å^3^	

### Data collection

**Table:**

Bruker SMART APEX CCD diffractometer	2836 reflections with *I* > 2σ(*I*)
Radiation source: fine-focus sealed tube	*R*_int_ = 0.084
φ and ω scans	θ_max_ = 25.0°, θ_min_ = 1.9°
Absorption correction: multi-scan (*SADABS*; Sheldrick, 2004)	*h* = −9→9
*T*_min_ = 0.696, *T*_max_ = 0.736	*k* = −12→12
6808 measured reflections	*l* = −13→13
3262 independent reflections	

### Refinement

**Table:**

Refinement on *F*^2^	Primary atom site location: structure-invariant direct methods
Least-squares matrix: full	Secondary atom site location: difference Fourier map
*R*[*F*^2^ > 2σ(*F*^2^)] = 0.036	Hydrogen site location: inferred from neighbouring sites
*wR*(*F*^2^) = 0.097	H atoms treated by a mixture of independent and constrained refinement
*S* = 1.03	*w* = 1/[σ^2^(*F*_o_^2^) + (0.0593*P*)^2^] where *P* = (*F*_o_^2^ + 2*F*_c_^2^)/3
3262 reflections	(Δ/σ)_max_ = 0.001
278 parameters	Δρ_max_ = 0.76 e Å^−^^3^
18 restraints	Δρ_min_ = −0.57 e Å^−^^3^

### Special details

**Table:**

Geometry. All e.s.d.'s (except the e.s.d. in the dihedral angle between two l.s. planes) are estimated using the full covariance matrix. The cell e.s.d.'s are taken into account individually in the estimation of e.s.d.'s in distances, angles and torsion angles; correlations between e.s.d.'s in cell parameters are only used when they are defined by crystal symmetry. An approximate (isotropic) treatment of cell e.s.d.'s is used for estimating e.s.d.'s involving l.s. planes.
Refinement. Refinement of *F*^2^ against ALL reflections. The weighted *R*-factor *wR* and goodness of fit *S* are based on *F*^2^, conventional *R*-factors *R* are based on *F*, with *F* set to zero for negative *F*^2^. The threshold expression of *F*^2^ > σ(*F*^2^) is used only for calculating *R*-factors(gt) *etc*. and is not relevant to the choice of reflections for refinement. *R*-factors based on *F*^2^ are statistically about twice as large as those based on *F*, and *R*- factors based on ALL data will be even larger.

### Fractional atomic coordinates and isotropic or equivalent isotropic displacement parameters (Å^2^)

**Table:**

	*x*	*y*	*z*	*U*_iso_*/*U*_eq_	
Mg1	0.5000	1.0000	0.5000	0.0274 (3)	
Cu1	0.53658 (3)	0.70272 (3)	0.98705 (2)	0.02798 (13)	
C1	0.7984 (3)	0.5135 (2)	0.8983 (2)	0.0278 (5)	
C2	0.9093 (3)	0.4662 (2)	0.8009 (2)	0.0318 (5)	
H2	0.9106	0.5116	0.7219	0.038*	
C3	1.0174 (3)	0.3528 (2)	0.8201 (3)	0.0338 (6)	
C4	1.0200 (3)	0.2837 (2)	0.9381 (3)	0.0378 (6)	
H4	1.0947	0.2089	0.9510	0.045*	
C5	0.9110 (3)	0.3276 (2)	1.0342 (3)	0.0357 (6)	
H5	0.9120	0.2808	1.1124	0.043*	
C6	0.7962 (3)	0.4422 (2)	1.0192 (2)	0.0297 (5)	
C7	0.6919 (3)	0.4804 (2)	1.1269 (2)	0.0311 (5)	
H7	0.7045	0.4283	1.2012	0.037*	
C8	0.4821 (3)	0.6142 (3)	1.2436 (2)	0.0354 (6)	
H8A	0.5625	0.6201	1.2940	0.043*	
H8B	0.4077	0.5488	1.2875	0.043*	
C9	0.3683 (3)	0.7412 (2)	1.2172 (2)	0.0289 (5)	
C10	0.2895 (3)	0.9088 (2)	1.0529 (2)	0.0319 (5)	
H10A	0.3256	0.9790	1.0762	0.038*	
H10B	0.1642	0.9065	1.0844	0.038*	
C11	0.3290 (3)	0.9256 (2)	0.9147 (2)	0.0308 (5)	
C12	1.1256 (4)	0.3610 (3)	0.6070 (3)	0.0504 (7)	
H12A	1.0081	0.3695	0.5930	0.076*	
H12B	1.2029	0.3108	0.5540	0.076*	
H12C	1.1643	0.4432	0.5911	0.076*	
N1	0.5819 (3)	0.58115 (19)	1.12892 (18)	0.0301 (4)	
N2	0.3860 (3)	0.79083 (19)	1.10172 (18)	0.0315 (5)	
O1	0.6989 (2)	0.62339 (15)	0.87217 (15)	0.0315 (4)	
O3	0.4460 (2)	0.84413 (16)	0.86727 (15)	0.0352 (4)	
O4	0.2500 (2)	1.01549 (17)	0.85495 (16)	0.0434 (5)	
O2	0.2727 (2)	0.78645 (17)	1.30527 (15)	0.0370 (4)	
O5	1.1276 (2)	0.30083 (17)	0.73004 (18)	0.0458 (5)	
O6	0.4664 (2)	1.10040 (18)	0.63586 (15)	0.0376 (4)	
H6A	0.553 (3)	1.120 (3)	0.656 (3)	0.056*	
H6B	0.394 (3)	1.077 (3)	0.702 (2)	0.056*	
O7	0.2365 (2)	1.0350 (2)	0.49409 (17)	0.0423 (5)	
H7A	0.159 (4)	1.017 (3)	0.557 (2)	0.063*	
H7B	0.194 (4)	1.096 (2)	0.448 (2)	0.063*	
O8	0.4514 (3)	0.83655 (18)	0.62765 (16)	0.0423 (5)	
H8C	0.393 (4)	0.785 (3)	0.616 (3)	0.063*	
H8D	0.442 (5)	0.836 (3)	0.7002 (18)	0.063*	
O9	0.8995 (3)	0.7896 (2)	0.68699 (18)	0.0479 (5)	
H9A	0.908 (5)	0.855 (2)	0.712 (3)	0.072*	
H9B	0.837 (4)	0.742 (3)	0.737 (3)	0.072*	
O10	1.0000 (3)	0.9857 (2)	0.28950 (19)	0.0462 (5)	
H10C	1.072 (4)	0.922 (2)	0.298 (3)	0.069*	
H10D	0.934 (4)	0.984 (3)	0.246 (3)	0.069*	
O11	0.2361 (3)	0.6905 (2)	0.56744 (19)	0.0483 (5)	
H11A	0.145 (3)	0.724 (3)	0.602 (3)	0.072*	
H11B	0.249 (4)	0.708 (3)	0.4918 (16)	0.072*	

### Atomic displacement parameters (Å^2^)

**Table:**

	*U*^11^	*U*^22^	*U*^33^	*U*^12^	*U*^13^	*U*^23^
Mg1	0.0289 (6)	0.0339 (6)	0.0190 (5)	−0.0054 (4)	−0.0024 (4)	−0.0058 (4)
Cu1	0.03038 (19)	0.03127 (19)	0.01925 (18)	0.00204 (12)	−0.00343 (12)	−0.00437 (12)
C1	0.0263 (11)	0.0252 (11)	0.0331 (13)	−0.0035 (9)	−0.0082 (10)	−0.0054 (10)
C2	0.0310 (12)	0.0309 (12)	0.0332 (14)	−0.0027 (10)	−0.0070 (10)	−0.0061 (10)
C3	0.0276 (12)	0.0311 (12)	0.0450 (15)	−0.0024 (10)	−0.0071 (11)	−0.0135 (11)
C4	0.0351 (13)	0.0284 (12)	0.0506 (17)	0.0032 (10)	−0.0149 (12)	−0.0075 (11)
C5	0.0374 (13)	0.0292 (12)	0.0409 (15)	−0.0032 (10)	−0.0161 (12)	−0.0003 (11)
C6	0.0287 (12)	0.0280 (12)	0.0338 (13)	−0.0039 (9)	−0.0105 (10)	−0.0042 (10)
C7	0.0321 (12)	0.0335 (13)	0.0275 (13)	−0.0082 (10)	−0.0113 (10)	0.0023 (10)
C8	0.0336 (13)	0.0496 (15)	0.0211 (12)	−0.0027 (11)	−0.0070 (10)	−0.0031 (11)
C9	0.0267 (11)	0.0408 (13)	0.0230 (12)	−0.0097 (10)	−0.0048 (9)	−0.0104 (10)
C10	0.0386 (13)	0.0327 (13)	0.0226 (12)	−0.0009 (10)	−0.0006 (10)	−0.0096 (10)
C11	0.0338 (13)	0.0300 (12)	0.0263 (13)	−0.0012 (10)	−0.0015 (10)	−0.0070 (10)
C12	0.0512 (17)	0.0503 (17)	0.0473 (18)	0.0053 (13)	−0.0041 (14)	−0.0182 (14)
N1	0.0284 (10)	0.0366 (11)	0.0248 (10)	−0.0029 (8)	−0.0071 (8)	−0.0038 (8)
N2	0.0365 (11)	0.0345 (11)	0.0217 (10)	−0.0004 (9)	−0.0042 (9)	−0.0062 (8)
O1	0.0340 (9)	0.0305 (8)	0.0250 (9)	0.0041 (7)	−0.0038 (7)	−0.0033 (7)
O3	0.0448 (10)	0.0347 (9)	0.0193 (9)	0.0084 (7)	−0.0018 (7)	−0.0053 (7)
O4	0.0514 (11)	0.0391 (10)	0.0290 (10)	0.0146 (8)	−0.0035 (8)	−0.0040 (8)
O2	0.0370 (9)	0.0524 (11)	0.0215 (9)	−0.0036 (8)	−0.0024 (7)	−0.0116 (8)
O5	0.0446 (11)	0.0409 (10)	0.0486 (12)	0.0102 (8)	−0.0060 (9)	−0.0161 (9)
O6	0.0400 (10)	0.0481 (11)	0.0269 (9)	−0.0122 (8)	0.0019 (8)	−0.0154 (8)
O7	0.0303 (9)	0.0610 (12)	0.0288 (10)	−0.0006 (8)	−0.0027 (8)	−0.0019 (9)
O8	0.0591 (12)	0.0455 (11)	0.0240 (9)	−0.0185 (9)	−0.0083 (9)	−0.0016 (8)
O9	0.0487 (12)	0.0496 (12)	0.0385 (12)	−0.0082 (9)	−0.0032 (9)	0.0015 (9)
O10	0.0365 (11)	0.0617 (13)	0.0366 (11)	0.0040 (9)	−0.0026 (8)	−0.0140 (10)
O11	0.0530 (12)	0.0529 (12)	0.0393 (12)	−0.0108 (10)	−0.0093 (10)	−0.0067 (10)

### Geometric parameters (Å, °)

**Table:**

Mg1—O7^i^	2.0591 (18)	C8—H8A	0.9700
Mg1—O7	2.0591 (18)	C8—H8B	0.9700
Mg1—O6	2.0598 (17)	C9—O2	1.266 (3)
Mg1—O6^i^	2.0599 (17)	C9—N2	1.302 (3)
Mg1—O8	2.0625 (18)	C10—N2	1.451 (3)
Mg1—O8^i^	2.0626 (18)	C10—C11	1.526 (3)
Cu1—O1	1.8797 (17)	C10—H10A	0.9700
Cu1—N2	1.892 (2)	C10—H10B	0.9700
Cu1—N1	1.920 (2)	C11—O4	1.231 (3)
Cu1—O3	1.9799 (16)	C11—O3	1.292 (3)
C1—O1	1.336 (3)	C12—O5	1.422 (4)
C1—C2	1.401 (3)	C12—H12A	0.9600
C1—C6	1.430 (3)	C12—H12B	0.9600
C2—C3	1.389 (3)	C12—H12C	0.9600
C2—H2	0.9300	O6—H6A	0.840 (17)
C3—O5	1.361 (3)	O6—H6B	0.851 (17)
C3—C4	1.400 (4)	O7—H7A	0.840 (17)
C4—C5	1.368 (4)	O7—H7B	0.833 (17)
C4—H4	0.9300	O8—H8C	0.831 (17)
C5—C6	1.422 (3)	O8—H8D	0.822 (17)
C5—H5	0.9300	O9—H9A	0.848 (18)
C6—C7	1.432 (4)	O9—H9B	0.810 (18)
C7—N1	1.288 (3)	O10—H10C	0.826 (18)
C7—H7	0.9300	O10—H10D	0.810 (17)
C8—N1	1.466 (3)	O11—H11A	0.813 (18)
C8—C9	1.533 (3)	O11—H11B	0.835 (18)
			
O7^i^—Mg1—O7	179.999 (1)	C9—C8—H8A	109.7
O7^i^—Mg1—O6	88.05 (8)	N1—C8—H8B	109.7
O7—Mg1—O6	91.95 (8)	C9—C8—H8B	109.7
O7^i^—Mg1—O6^i^	91.95 (8)	H8A—C8—H8B	108.2
O7—Mg1—O6^i^	88.05 (8)	O2—C9—N2	127.6 (2)
O6—Mg1—O6^i^	180.0	O2—C9—C8	119.1 (2)
O7^i^—Mg1—O8	90.55 (8)	N2—C9—C8	113.2 (2)
O7—Mg1—O8	89.45 (8)	N2—C10—C11	107.92 (19)
O6—Mg1—O8	90.60 (8)	N2—C10—H10A	110.1
O6^i^—Mg1—O8	89.40 (8)	C11—C10—H10A	110.1
O7^i^—Mg1—O8^i^	89.45 (8)	N2—C10—H10B	110.1
O7—Mg1—O8^i^	90.55 (8)	C11—C10—H10B	110.1
O6—Mg1—O8^i^	89.40 (8)	H10A—C10—H10B	108.4
O6^i^—Mg1—O8^i^	90.60 (8)	O4—C11—O3	123.8 (2)
O8—Mg1—O8^i^	180.000 (1)	O4—C11—C10	118.8 (2)
O1—Cu1—N2	175.66 (8)	O3—C11—C10	117.4 (2)
O1—Cu1—N1	96.90 (8)	O5—C12—H12A	109.5
N2—Cu1—N1	83.79 (9)	O5—C12—H12B	109.5
O1—Cu1—O3	95.95 (7)	H12A—C12—H12B	109.5
N2—Cu1—O3	83.51 (8)	O5—C12—H12C	109.5
N1—Cu1—O3	167.03 (8)	H12A—C12—H12C	109.5
O1—C1—C2	117.5 (2)	H12B—C12—H12C	109.5
O1—C1—C6	123.6 (2)	C7—N1—C8	121.6 (2)
C2—C1—C6	118.9 (2)	C7—N1—Cu1	124.53 (18)
C3—C2—C1	121.2 (2)	C8—N1—Cu1	113.87 (16)
C3—C2—H2	119.4	C9—N2—C10	124.0 (2)
C1—C2—H2	119.4	C9—N2—Cu1	119.48 (17)
O5—C3—C2	124.4 (2)	C10—N2—Cu1	116.46 (15)
O5—C3—C4	115.0 (2)	C1—O1—Cu1	125.02 (15)
C2—C3—C4	120.6 (2)	C11—O3—Cu1	114.46 (15)
C5—C4—C3	119.0 (2)	C3—O5—C12	118.7 (2)
C5—C4—H4	120.5	Mg1—O6—H6A	121 (2)
C3—C4—H4	120.5	Mg1—O6—H6B	118 (2)
C4—C5—C6	122.6 (2)	H6A—O6—H6B	106 (2)
C4—C5—H5	118.7	Mg1—O7—H7A	121 (2)
C6—C5—H5	118.7	Mg1—O7—H7B	124 (2)
C5—C6—C1	117.7 (2)	H7A—O7—H7B	108 (2)
C5—C6—C7	117.5 (2)	Mg1—O8—H8C	121 (2)
C1—C6—C7	124.7 (2)	Mg1—O8—H8D	120 (2)
N1—C7—C6	125.1 (2)	H8C—O8—H8D	112 (3)
N1—C7—H7	117.4	H9A—O9—H9B	113 (3)
C6—C7—H7	117.4	H10C—O10—H10D	114 (3)
N1—C8—C9	109.61 (19)	H11A—O11—H11B	114 (3)
N1—C8—H8A	109.7		

Symmetry codes: (i) −*x*+1, −*y*+2, −*z*+1.

### Hydrogen-bond geometry (Å, °)

**Table:**

*D*—H···*A*	*D*—H	H···*A*	*D*···*A*	*D*—H···*A*
O6—H6B···O4	0.85 (2)	1.91 (2)	2.755 (3)	175 (3)
O6—H6A···O2^ii^	0.84 (2)	2.02 (2)	2.836 (2)	165 (3)
O7—H7A···O10^i^	0.84 (2)	1.91 (2)	2.734 (3)	165 (3)
O7—H7B···O9^i^	0.83 (2)	1.96 (2)	2.776 (3)	166 (3)
O8—H8C···O11	0.83 (2)	1.98 (2)	2.797 (3)	169 (3)
O8—H8D···O3	0.82 (2)	1.96 (2)	2.775 (2)	173 (3)
O9—H9A···O10^iii^	0.85 (2)	1.99 (2)	2.787 (3)	157 (3)
O9—H9B···O1	0.81 (2)	2.01 (2)	2.816 (3)	174 (4)
O10—H10C···O2^iv^	0.83 (2)	1.99 (2)	2.805 (3)	171 (4)
O10—H10D···O4^i^	0.81 (2)	2.05 (2)	2.857 (3)	176 (4)
O11—H11A···O9^v^	0.81 (2)	2.05 (2)	2.857 (3)	172 (4)
O11—H11B···O2^vi^	0.84 (2)	2.10 (2)	2.927 (3)	170 (4)

Symmetry codes: (ii) −*x*+1, −*y*+2, −*z*+2; (i) −*x*+1, −*y*+2, −*z*+1; (iii) −*x*+2, −*y*+2, −*z*+1; (iv) *x*+1, *y*, *z*−1; (v) *x*−1, *y*, *z*; (vi) *x*, *y*, *z*−1.

## References

[bb1] Brandenburg, K. (2006). *DIAMOND* Crystal Impact GbR, Bonn, Germany.

[bb2] Bruker (2002). *SMART* Bruker AXS Inc., Madison, Wisconsin, USA.

[bb3] Bruker (2003). *SAINT-Plus* Bruker AXS Inc., Madison, Wisconsin, USA.

[bb4] Gupta, K. C., Sutar, A. K. & Lin, C. C. (2009). *Coord. Chem. Rev.* **253**, 1926–1946.

[bb5] Jiang, J., Lu, Y., Yuan, L. & Liu, W. (2009). *Acta Cryst.* E**65**, m952. 10.1107/S1600536809026750 PMC2977345 21583401

[bb6] Sakamoto, M., Manseki, K. & Okawa, H. (2001). *Coord. Chem. Rev.* **219**, 379–414.

[bb7] Sheldrick, G. M. (2004). *SADABS* University of Göttingen, Germany.

[bb8] Sheldrick, G. M. (2008). *Acta Cryst.* A**64**, 112–122. 10.1107/S0108767307043930 18156677

[bb9] Vigato, P. A. & Tamburini, S. (2008). *Coord. Chem. Rev.* **252**, 1871–1995.

[bb10] Vigato, P. A., Tamburini, S. & Bertolo, L. (2007). *Coord. Chem. Rev.* **251**, 1311–1492.

[bb11] Zhang, G., Ye, L., Zhang, Y. & Liu, W. (2008). *Acta Cryst.* E**64**, m94–m95. 10.1107/S1600536807063635 PMC2914968 21200661

